# Sphingosine-1-Phosphate Receptor and Kinase Expression in the Reproductive Tract Is Associated with HIV Infection and Preterm Birth in a Cohort of Pregnant Women in Zambia

**DOI:** 10.3390/v18050559

**Published:** 2026-05-14

**Authors:** Rachel S. Resop, Innocent Mwape, Yuri V. Sebastião, Katelyn J. Rittenhouse, Ntazana Sindano, Humphrey Mwape, Margaret P. Kasaro, Bellington Vwalika, Joan T. Price, Jeffrey S. A. Stringer, Kristina De Paris

**Affiliations:** 1 Department of Obstetrics and Gynecology, School of Medicine, University of North Carolina, Chapel Hill, NC 27599, USA; 2 National Institutes of Health, Bethesda, MD 20892, USA; 3 Centre for Infectious Disease Research, Lusaka 10101, Zambia; 4 UNC Global Projects—Zambia, LLC, Lusaka 10101, Zambia; 5 Obstetrics and Gynaecology, School of Medicine, University of Zambia, Lusaka 10101, Zambia; 6 Department of Microbiology and Immunology, School of Medicine, University of North Carolina, Chapel Hill, NC 27599, USA

**Keywords:** human immunodeficiency virus, preterm birth, sphingosine-1-phosphate, female reproductive tract, adverse birth outcomes, longitudinal pregnancy cohort

## Abstract

Women living with HIV face an increased burden of spontaneous preterm birth (sPTB); however, the underlying immunological mechanisms of sPTB and its association with HIV infection are poorly understood. Although the limited earlier literature implicates sphingosine-1-phosphate (S1P), a lysosphingolipid signaling molecule, in reproductive biology, the association of S1P signaling with HIV and sPTB has not been investigated. We examined whether two S1P signaling components, S1P receptors and sphingosine kinases, are expressed in the female reproductive tract and whether levels are associated with HIV status or spontaneous preterm birth. We quantified the mRNA expression of sphingosine-1-phosphate receptors 1 and 3 (*S1PR1*/*S1PR3*) and sphingosine kinases 1 and 2 (*SPHK1*/*SPHK2*) in 167 banked vaginal swab specimens collected between 14 and 26 weeks of gestation in a longitudinal pregnancy cohort in Lusaka, Zambia. We evaluated the expression of *S1PR1*, *S1PR3*, *SPHK1*, and *SPHK2* by real-time quantitative reverse transcription PCR (RT-qPCR) in four groups (n = 41–42 each): women without HIV (WWoH) with term birth (≥37 weeks of gestation; TB), WWoH with spontaneous preterm birth (<37 weeks of gestation, sPTB), women with HIV (WWH) with TB, and WWH with sPTB. We found that S1P receptors and sphingosine kinases are expressed in the female reproductive tract. *SPHK1* and *SPHK2* mRNA expression were generally comparable among women independent of HIV status or birth outcome, though *SPHK2* trended toward higher expression in women with HIV and women with sPTB. In contrast, *S1PR1* mRNA trended toward higher expression in WWH vs. WWoH overall, as well as in WWH vs. WWoH among women with sPTB. Similarly, *S1PR3* mRNA expression was greater in women with HIV than in women without HIV, and WWH, both with TB and sPTB, had higher *S1PR3* mRNA expression than WWoH with TB. Perturbations in *S1PR1* and *S1PR3* mRNA expression may be associated with inflammation related to HIV infection and spontaneous preterm birth, suggesting that further studies of S1P signaling in pregnancy, especially among women with HIV, are warranted.

## 1. Introduction

Globally, over 40 million people are living with human immunodeficiency virus (HIV), an infection for which there is currently no cure [1]. The virus can be controlled by lifelong antiretroviral therapy (ART), which has improved the lives of many people living with HIV. However, even with adherence to ART, pregnant women with HIV experience a greater incidence of adverse birth outcomes including preterm birth [2] than their HIV-negative counterparts, and HIV-exposed infants, even if uninfected, go on to experience greater morbidity and mortality [3,4,5]. The underlying immunological mechanisms of these risks for mothers and infants are poorly understood.

Host immune molecules, including sphingolipids, facilitate HIV infection of CD4 T cells [6,7]. Moreover, sphingolipids mediate the transfer of HIV-1 virions from monocyte-derived immature dendritic cells to autologous T cells and between CD4 T cells [6,8]. Sphingosine-1-phosphate (S1P) is a lysophospholipid intra- and intercellular signaling molecule well-established in inflammation and many disease states including cancer [9,10], yet its part in HIV-1 pathogenesis, its presence and role within the female reproductive tract (FRT), and its association with adverse pregnancy outcomes in the context of HIV infection are incompletely understood.

S1P signaling occurs in the vaginal epithelium and alterations in this pathway are associated with pelvic organ prolapse [11]. Further, S1P levels are elevated in the FRT of women with polycystic ovarian syndrome (PCOS) [12]. Yamamoto et al. found a positive association between increasing *SPHK1* protein levels and activity with greater gestational age and observed increased *S1PR1* turnover and signaling in the decidua of the placenta during later pregnancy (term). In the same study, *S1PR3* protein expression was also greater at term, suggesting an important role for these S1P signaling components in pregnancy [13]. Yet, there is a paucity of literature examining the role of S1P signaling in female reproductive immunology and no reports to our knowledge on S1P in the context of HIV-1 infection in the FRT.

We have demonstrated that S1P signaling is crucial for robust HIV-1 infection of primary human CD4 T cells [7], that HIV-1 in turn can alter S1PR levels [14], and that modulation of S1P can inhibit HIV-1 infection by several mechanisms [7]. Specifically, modulation of S1P receptors on primary human cells with the S1PR functional antagonist FTY720 inhibits HIV-1 infection of CD4 T cells; promotes an anti-inflammatory, quiescent, and less infection-permissive state [7]; and blocks trans-infection of CD4 T cells by autologous macrophages [15]. As viral shedding can occur in the FRT, even in women with suppressed viremia [16,17,18], it is important to determine the extent to which S1P signaling is altered at the FRT mucosa in the context of HIV, the potential association with preterm birth, and whether this pathway can be modulated to attenuate inflammation in future clinical interventions.

Intriguingly, S1P is produced by the bacteria *Bacteroides* and this bacterial-derived sphingolipid participates in host metabolism [19,20] and is crucial for the maintenance of host intestinal integrity [20]. In the FRT, a *Lactobacillus*-dominant, homogeneous microbiome promotes a less inflammatory environment [21], whereas more diverse microbial communities and an anaerobic microbiome predispose women to inflammation [22] and increase the risk of preterm birth [23]. In our Zambian pregnancy cohort, an anaerobic vaginal microflora is more common among women with HIV (WWH) than among HIV-uninfected women (women without HIV, WWoH) [24]. Thus, anaerobic species that produce S1P in the FRT might potentially differ by HIV serostatus as previously suggested [25].

Further, pregnant WWH have higher inflammation in the vaginal tract, but not in the plasma, than pregnant WWoH, and vaginal inflammation is associated with preterm birth [26]. As infection with HIV potentiates changes in the microbiome and is associated with persistent immune activation, S1P signaling may play a central role in both processes. As the modulation of S1PR and S1P in the FRT of WWH and HIV-negative women, as well as the association of these processes with preterm birth in WWH and WWoH, are largely unknown, we aimed to investigate (1) the presence of SPHK and S1PR in the FRT, and (2) the potential association of these S1P components with HIV infection and birth outcome.

We hypothesized that sphingosine-1-phosphate signaling in the reproductive tract of WWH differs from that of WWoH and that this disparity may be associated with spontaneous preterm birth. *S1PR1*, *S1PR3*, *SPHK1*, and SPHK2 were chosen as the S1P biomarkers for analysis in this pilot study due to the existing literature supporting their potential role in the FRT (e.g., Yamamoto et al. demonstrated that S1P signaling fluctuates in the placenta throughout gestation, with *SPHK1* expression, S1P receptor 1/3 signaling, and S1P turnover increasing concomitantly with increasing gestational age [13]). Moreover, the sphingolipid composition of the FRT may differ concordantly with alterations in the vaginal microflora and inflammatory microenvironment. Thus, understanding this system may lead to further studies aimed at modifying S1P signaling to address FRT inflammation and adverse birth outcomes.

## 2. Materials and Methods

### 2.1. Study Design

This investigation is a sub-study nested in the Zambia Preterm Birth Prevention Study (ZAPPS), an ongoing longitudinal pregnancy cohort based in Lusaka, Zambia, and the Improving Pregnancy Outcomes with Progesterone (IPOP) study, also based in Lusaka [27,28]. These studies drew from the same catchment populations, were conducted at the same facilities, and shared most protocol elements, including antenatal assessments, visit timing, and a standardized procedure for phenotyping adverse birth outcomes. All participants had a viable intrauterine pregnancy confirmed by ultrasonography and underwent standardized ultrasound dating using Intergrowth 21st formulas [29,30]. Sampling from the above parent studies, we selected n = 42 women with HIV (WWH) with spontaneous preterm birth (sPTB, <37 weeks of gestation), then randomly selected n = 42 women without HIV (WWoH) with term birth (TB, ≥37 weeks of gestation), n = 42 WWoH with sPTB, and n = 42 WWH with TB. One specimen was not processed due to technical issues in the lab, resulting in a total of 167 specimens processed across the four groups (n = 41–42 each). As our outcome of interest was spontaneous preterm birth and sPTB cases occur much less frequently than TB, this design allowed selection of women who had already experienced sPTB rather than waiting for birth outcomes to occur. Sample size was determined based on the available budget for this pilot project, accounting for the projected cost of laboratory analysis of four S1P signaling parameters. Given the pre-determined sample size of n = 42 per group, we expected a priori to have a power of 0.8 to detect a minimum mean difference of approx. 10–14% in S1PR or SPHK expression in a two-sample test at an alpha of 0.05, assuming a relative standard deviation of approx. 22.5% based on our previous S1P signaling studies [15]. The characteristics of the participant population are summarized in Table 1.

Inclusion criteria included HIV+ or HIV− and sPTB or TB singleton pregnancies among participants in the ZAPPS/IPOP studies. We excluded cases of provider-initiated preterm birth (including medically indicated preterm delivery for maternal or fetal indications such as preeclampsia, fetal growth restriction, or non-reassuring fetal status) because these outcomes are frequently driven by distinct clinical pathophysiologic processes that may independently alter inflammatory signaling pathways, including S1P-related mechanisms. Inclusion of medically indicated cases could therefore introduce heterogeneity and confounding unrelated to spontaneous labor biology, obscuring potential associations between S1P signaling, HIV-related mucosal immune activation, and spontaneous preterm birth. Restricting the analysis to spontaneous preterm birth allowed us to more specifically examine biological pathways linked to inflammation and HIV in the context of preterm labor.

We leveraged the comprehensive ZAPPS and IPOP sample repositories and datasets to analyze stored frozen vaginal swab specimens collected between 14 and 26 weeks of gestational age for four S1P biomarkers and their association with HIV status and birth outcome (distribution of gestational age at sample collection vs. S1P biomarkers, Supplemental Figure S1). Specimen collection and laboratory analysis took place onsite at our clinical center and laboratory at the University Teaching Hospital (UTH) in Lusaka, as well as in collaboration with the Centre for Infectious Disease Research in Zambia (CIDRZ), also based in Lusaka. Using real-time quantitative reverse-transcription PCR (RT-qPCR), we evaluated the expression of two of the five S1P receptors (*S1PR1* and *S1PR3*) and the two isoforms of sphingosine kinase, the enzyme that phosphorylates sphingosine to generate the bioactive signaling molecule S1P (*SPHK1* and *SPHK2*).

### 2.2. Human Subjects

This work was approved under the ZAPPS and IPOP Protocols by the University of North Carolina Institutional Review Board (Study No. 16-2174 and 17-1173, approval date 9 June 2025) and the University of Zambia Biomedical Research Ethics committee (Ref. No. 004-09-16, approval date 3 November 2025).

### 2.3. Specimen Collection

Mid-vaginal dry polyester swabs were collected between 14 and 26 weeks of gestation (at either enrollment or the second antenatal visit) and were stored on-site at −80 °C.

### 2.4. Assessment of S1PR and SPHK Levels in the FRT

We quantified the mRNA expression of sphingosine-1-phosphate receptors 1 and 3 (*S1PR1*/*S1PR3*) and sphingosine kinases 1 and 2 (*SPHK1*/*SPHK2*) in stored frozen vaginal swab specimens collected between 14 and 26 weeks of gestational age by real-time quantitative reverse transcription PCR (RT-qPCR) following isolation of RNA from vaginal swab specimens with the RNeasy Plus Mini isolation kit (Qiagen, Venlo, The Netherlands, Cat#74136) and RNA quantification (Qubit instrument and reagents, Thermo Fisher Scientific, Cat#Q33239). RT-qPCR was performed as previously done [31]. All PCR reagents and equipment were from Thermo Fisher Scientific, Waltham, MA, USA, including Taqman primer/probe kits for *S1PR1/3* and *SPHK1/2* (FAM, Cat#4331182, assay IDs Hs05021992_s1, Hs00245464_s1, Hs00184211_m1, and Hs01016543_g1, respectively) and *GAPDH* housekeeping gene control (VIC, Cat#4448490, assay ID Hs02786624_g1), Taqman Superscript III 1 Step Platinum Taq PCR reagents (Cat#11732088), and the ABI 7500 Fast Real-Time PCR system thermal cycler (Cat#4351106) and companion software. We analyzed RT-qPCR data by the delta-delta CT relative expression method [32] in Microsoft Excel and summarized results in GraphPad Prism software (version 10.2.0). To be more intuitive for the reader, we present the negative values of the actual delta CT values as lower CT (threshold) values correspond to greater gene expression while higher CT values correspond to lower gene expression.

### 2.5. Analysis

We analyzed the association between S1P parameters (levels of *S1PR*/*SPHK*) and sPTB within HIV+/− strata by unpaired two-sided Mann–Whitney test and non-parametric test with multiple comparisons (Kruskal–Wallis) in Graphpad Prism software. In this exploratory study, reported *p*-values are unadjusted for multiple testing.

## 3. Results

**Figure 1:**
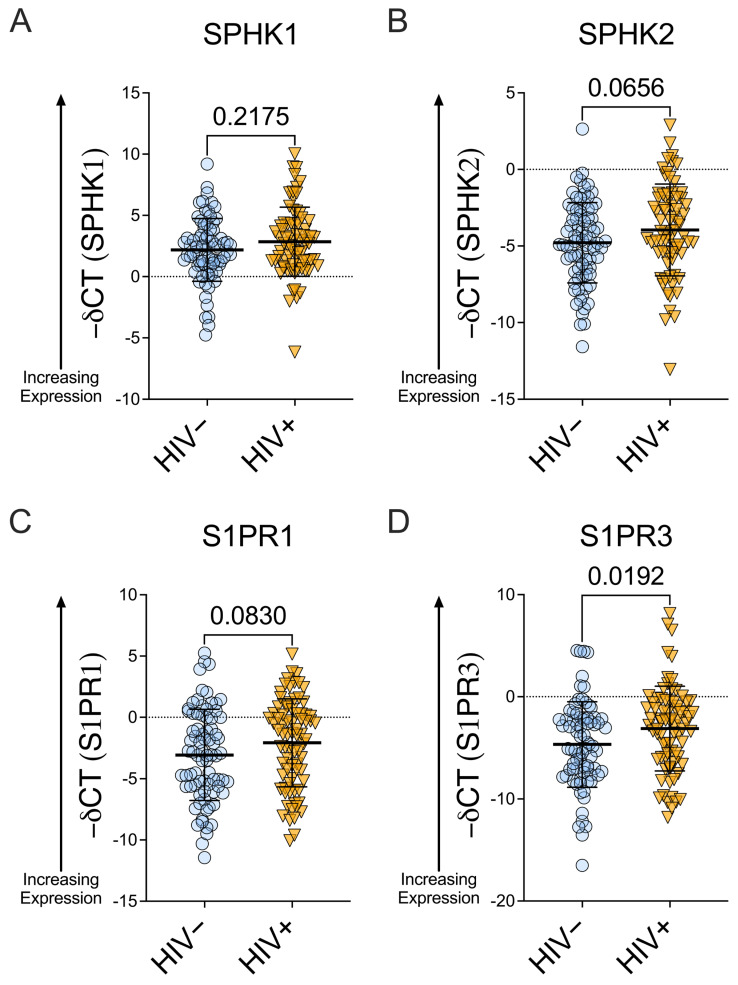

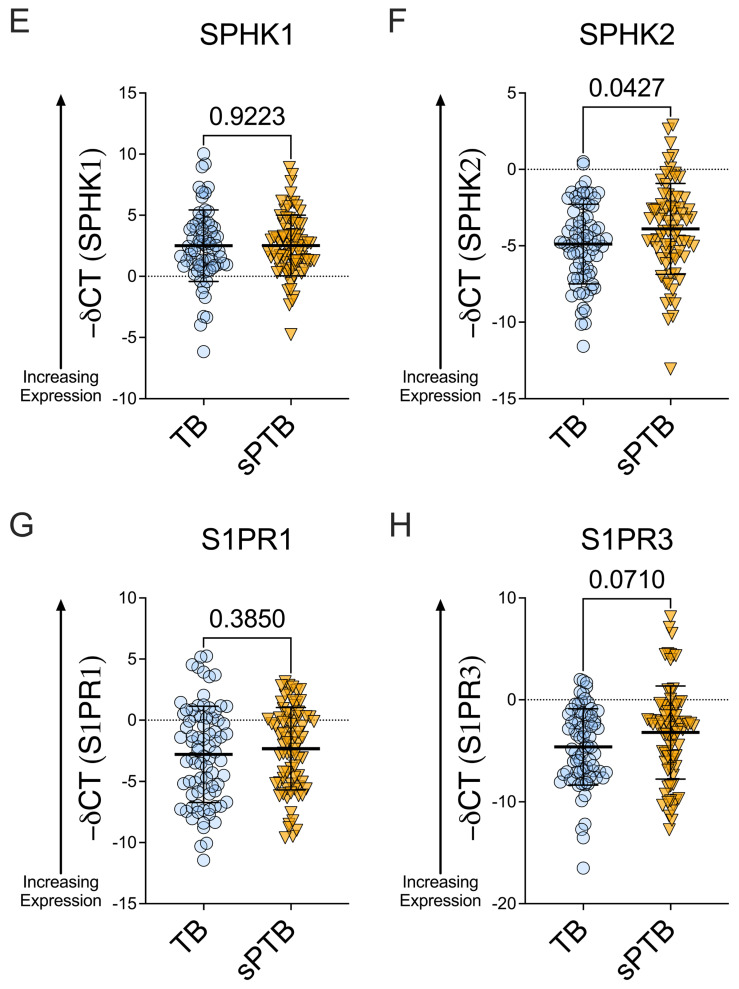
Expression of *SPHK1/2* and *S1PR1/3* in women with and without HIV and with full-term or preterm birth. mRNA expression levels of *SPHK1*, *SPHK2*, *S1PR1*, and *S1PR3* were assessed by RT-qPCR and compared between women with HIV (WWH) and women without HIV (WWoH) and between women with spontaneous preterm birth (sPTB) and women with term birth (TB). We present negative ∆CT values as lower CT values correspond to greater S1P receptor or sphingosine kinase expression, and vice versa. Higher values on the y-axis represent higher mRNA expression (upward arrow for ease of understanding). (**A**): Among WWH, *SPHK1* mRNA expression was comparable to levels in WWoH (*p* = 0.2175, Mann–Whitney). (**B**): *SPHK2* mRNA expression trended higher among WWH vs. WWoH (*p* = 0.0656, Mann–Whitney). (**C**): *S1PR1* expression trended higher among WWH vs. WWoH (approx. 2-fold greater in WWH, *p* = 0.0830, Mann–Whitney). (**D**): *S1PR3* levels were higher among WWH as compared to WWoH (approx. 2.95-fold, *p* = 0.0192, Mann–Whitney). (**E**): Among women with sPTB, *SPHK1* mRNA expression was not statistically significantly different compared to women with TB (*p* = 0.9223, Mann–Whitney). (**F**): *SPHK2* expression trended higher among women with sPTB vs women with TB (*p* = 0.0427, Mann–Whitney). (**G**): *S1PR1* levels were comparable among women with sPTB and TB (*p* = 0.3850, Mann–Whitney). (**H**): *S1PR3* levels trended higher among women with sPTB vs women with TB (*p* = 0.0710, Mann–Whitney). Mean + SD shown.

S1P receptors and sphingosine kinases are expressed in the female reproductive tract. Among women with HIV, *SPHK1* and *SPHK2* mRNA expression were comparable to levels observed among women without HIV, though *SPHK2* mRNA expression trended higher among WWH vs. WWoH (*SPHK1*: mean −deltaCT [∆CT ] = 2.18 for WWoH and 2.85 for WWH; *SPHK2*: mean −∆CT = −4.79 for WWoH and −3.95 for WWH; *p* = 0.2175 for *SPHK1* and *p* = 0.0656 for *SPHK2*, Mann–Whitney; Figure 1A,B). *S1PR1* expression trended higher in WWH than in WWoH (approx. 2-fold (2^−∆CT^); mean −∆CT = −3.07 for WWoH and −2.08 for WWH; *p* = 0.0830, Mann–Whitney; Figure 1C), and *S1PR3* levels were approx. 2.95-fold higher (2^−∆CT^) in WWH than in WWoH (mean −∆CT = −4.67 for WWoH and −3.11 for WWH, *p* = 0.0192, Mann–Whitney; Figure 1D). Among women with sPTB, *SPHK1* mRNA levels were similar to levels of women with TB, while *SPHK2* trended higher among women with sPTB (*SPHK1*: mean −∆CT = 2.50 for TB and 2.52 for sPTB; *SPHK2*: mean −∆CT = −4.89 for TB and −3.89 for sPTB; *p* = 0.9223 and *p* = 0.0427 for *SPHK1* and *SPHK2* respectively, Mann–Whitney; Figure 1E,F). Similarly, *S1PR1* and *S1PR3* levels were comparable among women with TB and sPTB, though *S1PR3* trended higher in women with sPTB (*S1PR1*: mean −∆CT = −2.79 for TB and −2.33 for sPTB; *S1PR3*: mean −∆CT = −4.62 for TB and −3.20 for sPTB (an approx. 2.7-fold difference, 2^−∆CT^); *p* = 0.3850 and *p* = 0.0710, Mann–Whitney; Figure 1G,H).

**Figure 2:**
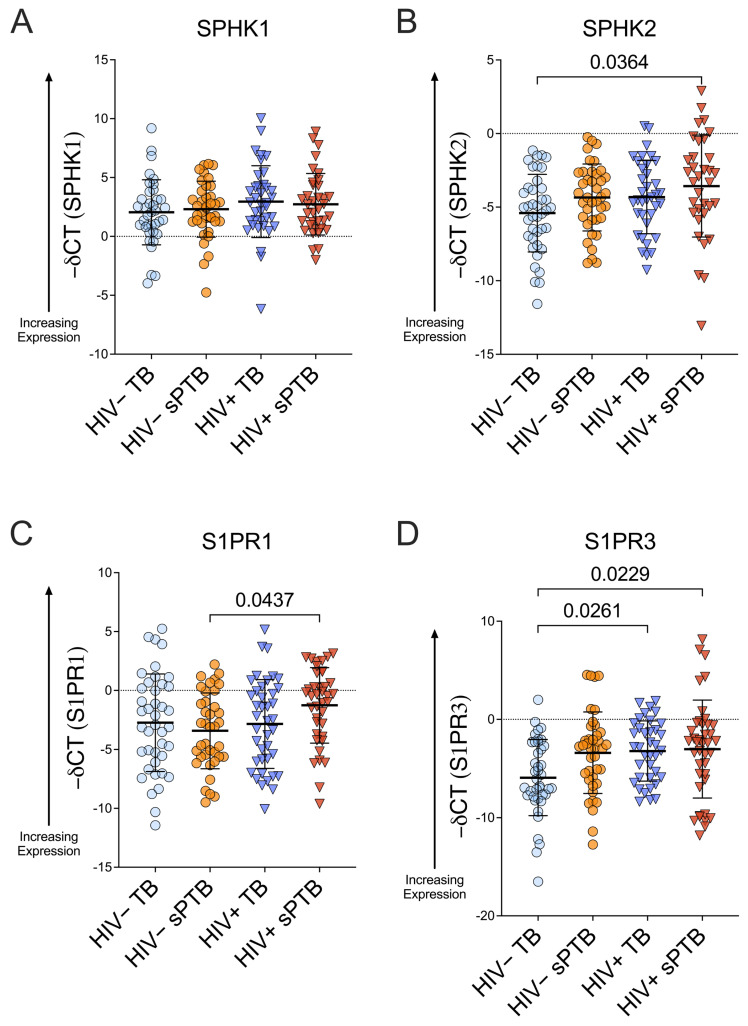
Expression of *SPHK1/2* and *S1PR1/3* in stratified populations: HIV− TB, HIV− sPTB, HIV+ TB, and HIV+ sPTB. mRNA expression levels of *SPHK1*, *SPHK2*, *S1PR1*, and *S1PR3* were assessed by RT-qPCR and compared when stratifying by HIV status and birth outcome. Negative ∆CT values are presented as lower CT values correspond to greater S1P receptor or sphingosine kinase mRNA expression. Higher values on the y-axis represent higher mRNA expression. (**A**): *SPHK1* expression was comparable among women across all HIV and birth outcome strata, with all comparisons for *SPHK1* resulting in non-significant differences. (**B**): *SPHK2* mRNA expression was greater in WWH with sPTB than in WWoH with TB (*p* = 0.0364, Kruskal–Wallis), with all other comparisons for SPHK2 resulting in non-significant differences. (**C**): *S1PR1* levels were elevated in women with HIV versus WWoH among all women with sPTB (*p* = 0.0437, Kruskal–Wallis) and trended higher among women with sPTB as compared to those with TB among all WWH. (**D**): Among WWoH, *S1PR3* levels trended higher in women with sPTB, and among all women with TB, *S1PR3* levels were statistically significantly higher in WWH (*p* = 0.0261, Kruskal–Wallis). Further, *S1PR3* levels were higher in WWH with sPTB as compared to WWoH and TB (*p* = 0.0229; Kruskal–Wallis).

When stratifying by HIV status and birth outcome, notable variability was observed in *SPHK* and *S1PR* levels across the four strata. *SPHK1* remained comparable among women across all HIV and birth outcome strata (mean −∆CT = 2.05 for HIV− TB, 2.31 for HIV− sPTB, 2.96 for HIV+ TB, and 2.73 for HIV+ sPTB, Figure 2A), and *SPHK2* mRNA expression was also largely stable, with the exception of higher expression in WWH with sPTB than in WWoH with TB (mean −∆CT = −5.41 for HIV− TB, −4.34 for HIV− sPTB, −4.32 for HIV+ TB, and −3.57 for HIV+ sPTB, *p* = 0.0364, WWH with sPTB vs. WWoH with TB, Kruskal–Wallis, Figure 2B).

In our stratified analysis, *S1PR1* mRNA expression was elevated in women with HIV versus WWoH among all women with sPTB (mean −∆CT = −2.73 for HIV− TB, −3.42 for HIV− sPTB, −2.84 for HIV+ TB, and −1.27 for HIV+ sPTB; *p* = 0.0437 for HIV+ sPTB vs. HIV− sPTB, Kruskal–Wallis, Figure 2C) and trended toward higher expression among women with sPTB as compared to those with TB among all WWH (Figure 2C). Among all women with TB, *S1PR3* levels were higher in WWH (mean −∆CT = −5.92 for HIV− TB, −3.39 for HIV− sPTB, −3.22 for HIV+ TB, and −3.02 for HIV+ sPTB; *p* = 0.0261 for HIV+ TB vs. HIV− TB, Kruskal–Wallis, Figure 2D). Further, *S1PR3* levels were higher in WWH with sPTB as compared to WWoH with TB (*p* = 0.0229, Kruskal–Wallis, Figure 2D).

In sum, in our pregnancy cohort, *SPHK1* and *SPHK2* mRNA expression were generally comparable among women independent of HIV status or birth outcome, though *SPHK2* trended toward higher expression in women with HIV and women with sPTB. In contrast, *S1PR1* and *S1PR3* mRNA expression varied by HIV status and, in stratified analyses, by birth outcome. The most consistent differences were observed by HIV status, with elevated *S1PR1* and *S1PR3* expression among women with HIV, while associations with spontaneous preterm birth were more modest and context-dependent.

## 4. Discussion

Among the 167 women in our pregnancy cohort, *SPHK1/2* levels were generally comparable among women irrespective of HIV status and birth outcome, although *SPHK2* levels were elevated in women with sPTB vs. women with TB overall, and in WWH who experienced sPTB versus WWoH with TB in stratified analysis. *S1PR1* and *S1PR3* mRNA expression varied by both parameters we investigated, HIV status and birth outcome. With respect to HIV status, women with HIV and preterm birth had higher (*p* = 0.0429) *S1PR1* mRNA levels than women without HIV and sPTB. Similarly, among all women, WWH had nearly 3-fold higher *S1PR3* mRNA expression (approx. 2.95-fold, *p* = 0.0192) than WWoH. WWH, both with term and preterm birth, had higher *S1PR3* mRNA expression than WWoH with TB (*p* = 0.0261 and *p* = 0.0229, respectively).

A major strength of this pilot investigation into sphingosine kinase and S1P receptor expression in the FRT is our ability to leverage a large, well-characterized longitudinal pregnancy cohort with extensive clinical data available. This allowed the study of the outcome of interest, spontaneous preterm birth, both among women living with HIV and women without HIV. To our knowledge, this is the first investigation of S1PR and SPHK in the FRT in the context of HIV and spontaneous preterm birth.

This study has several limitations, such as cross-sectional mid-pregnancy sampling, which precludes analysis of how S1P biomarkers may change across pregnancy. Another notable limitation is bulk tissue RNA isolation, which limits understanding of how *S1PR1/3* and *SPHK1/2* may differ across cell type and which cellular populations are responsible for their expression (e.g., whether the *S1PR/SPHK* differences observed reflect mucosal epithelia changes vs. alterations in infiltrating lymphocytes/immune cell recruitment). Although we did not perform cell-based assays such as flow cytometry due to resource limitations in this pilot study, based on the existing literature it is likely that differences in *S1PR/SPHK* levels are due to production by vaginal epithelial cells, and to a lesser extent, infiltrating lymphocytes (macrophages, neutrophils, and CD4 T cells) [11,12,13].

Another limitation is the lack of protein-level S1P receptor/sphingosine kinase measurements, as mRNA expression may not directly reflect protein levels, although the magnitude of the fold changes observed, especially for *S1PR3*, suggests that a functional protein difference is likely present (further studies are needed to validate this). Additionally, the inability to adjust for ART duration or viral suppression, which could have influenced expression levels of inflammatory cytokines and/or *S1PR/SPHK*, due to the nature of the descriptive analysis (comparison across four study groups), is a limitation.

Limitations in the existing literature on S1P signaling and female reproductive immunology present challenges in interpreting the results of our study, but potential contributing factors to the results we observed include the linkage of S1P signaling with inflammation related to HIV infection, including viral shedding in the FRT and the altered inflammatory cytokines that accompany these pathologies, and/or alterations in S1P signaling across gestational age (and thus, spontaneous preterm birth). As sPTB is a multifactorial outcome, further investigations into the interconnected nature of S1P signaling and fluctuations in inflammatory cytokines in the context of HIV infection and sPTB are needed.

We and others have previously reported increased inflammation in the FRT in WWH and that altered inflammatory cytokine levels in HIV-1 infection can impact S1P signaling. Earlier work from our group showed that women with HIV not on ART have higher vaginal inflammation than women without HIV, and vaginal inflammation (but not systemic inflammation) is associated with sPTB [26]. Previously, we investigated *S1PR1* changes during HIV infection *in vivo* and observed that increased *S1PR1* mRNA and protein expression during HIV infection is linked to increases in levels of inflammatory cytokines including IFN-α and TNF-α [14]. In our Zambian study population, vaginal inflammation predicts sPTB in multivariate analysis [26] and others have reported that IL-1β and IL-6 are associated with sPTB [33]. IL-1β increases *SPHK1* mRNA expression and can therefore increase S1P levels [34], which could further promote inflammatory signaling and production of pro-inflammatory cytokines including TNF-α. Thus, HIV-induced modifications to the cytokine signaling network may promote increased S1P signaling and ongoing inflammation, which could contribute to adverse birth outcomes.

Earlier investigations have shown that components of S1P signaling (S1PR3 and sphingosine lyase, a proxy measurement of S1P turnover) increase with increasing gestational age [13,35]. This suggests that the higher trending *S1PR3* expression in WWoH with spontaneous preterm birth as compared to WWoH with term birth, and the increased *S1PR3* expression in WWH with sPTB compared to WWoH with TB, may reflect heightened S1P signaling associated with preterm labor. In this pilot study we are unable to determine the underlying mechanisms of the observed *SPHK/S1PR* differences, so it is possible that either a premature activation of late pregnancy signaling or an inflammatory response could underpin these observations. *S1PR3* expression may also be increased due to HIV-related inflammation in the FRT, as we observed increased expression in WWH with term birth relative to WWoH and term birth. S1P receptor expression is upregulated during HIV infection of CD4 T cells [14], but whether this is also the case in the FRT in WWH has not been examined.

We found notable variation in *SPHK* and *S1PR* expression among the women in our study, especially in *SPHK2* and *S1PR3* levels in WWH who experienced preterm birth (Figure 2). We hypothesized that this variation may be due to differences in inflammation in WWH with sPTB, perhaps related to ART regimen/time on ART, viral load, or CD4 levels. We performed preliminary analyses to examine possible correlations between viral load and/or CD4 and *S1PR/SPHK* levels and did not observe a statistically significant correlation between either viral load or CD4 count and *SPHK1/2* or *S1PR1/3* mRNA expression in this cohort of women with HIV (simple linear regression, Supplemental Figures S2 and S3). This may suggest that variation in *SPHK*/*S1PR* levels in WWH and women with preterm birth is multifactorial, likely involving altered expression of inflammatory cytokines; thus it would be important in future work to probe whether inflammatory changes preclude both *SPHK*/*S1PR* changes and sPTB, or, alternately, whether there is a mechanistic link between sphingosine-1-phosphate signaling and preterm birth. In future studies we hope to pursue a greater understanding of the mechanism of variation in sphingosine kinase and S1P receptor expression, with the goal of applying this understanding to earlier identification of women who may be at increased risk of an adverse birth outcome, both among WWH and WWoH. Measurement of vaginal S1P and inflammatory cytokine levels in matched stored vaginal samples from WWH and WWoH in this pregnancy cohort could provide insight into the inflammatory environment of the FRT and allow analysis of the association of differences in these markers with S1PR and SPHK levels, HIV status, and birth outcomes. 

An additional area of interest is the expression of S1P receptors 1–5 (*S1PR1-5*) and *SPHK1/2* in banked placenta specimens collected at delivery. As earlier studies reporting changes in S1P signaling with gestational age were done in the placenta [13] these investigations would greatly enhance our understanding of the inflammatory changes experienced by WWH and women with preterm birth. Analysis of S1P signaling changes in the vaginal mucosa in earlier pregnancy (e.g., second trimester) and comparison with matched placenta specimens collected at delivery would allow evaluation of whether changes in S1P biomarkers in the vagina might preclude changes in the placenta, which could contribute to mechanistic understanding that could inform earlier diagnosis. We also plan to delve deeper into the analysis of clinical/epidemiologic indicators (e.g., income, education, living situation) and viral load/CD4 levels obtained for WWH and WWoH in our pregnancy cohort to examine potential associations between psychological stress, inflammation, S1P signaling, and adverse birth outcomes.

Finally, further investigations into the association of the vaginal microbiome and S1P signaling changes are an intriguing avenue of investigation. In the FRT, diverse microbial communities and an anaerobic microbiome predispose women to inflammation [22] and increase the risk of sPTB [23]; in our pregnancy cohort, an anaerobic vaginal microflora is more common among WWH than among WWoH [24] and an anaerobe-rich, *Lactobacillus*-deficient vaginal microbiome is associated with higher inflammation and elevated risk of preterm birth, especially in WWH [36]. Earlier work from our group, which included women in the same parent study as some of the women in our cohort, showed that a more diverse, anaerobic, *Lactobacillus*-deficient vaginal microbiome is associated with an elevated risk of preterm birth, especially in women with HIV. Specifically, women with HIV have higher relative abundance of a novel *Gardnerella* metagenomic subspecies, and presence of this subspecies predicts preterm birth [36]. It is intriguing to consider whether shifts in the microbiome could predispose women to an increased proportion of S1P-producing anaerobes like *Bacteroides* and subsequently altered levels of S1P metabolites that could interact with endogenous S1P receptors (potentially resulting in modulation of S1PR in response to increased ligand production), signaling pathways, and/or feedback loops, possibly contributing to adverse birth outcomes.

## 5. Conclusions

In this study, we investigated the expression of various components of the S1P signaling pathway in the female reproductive tract, including two S1P receptors (*S1PR1/3*) and the two sphingosine kinase isoforms (*SPHK1/2*), and potential associations with HIV status and preterm birth. Although the limited prior literature suggested that S1P signaling changes with gestational age and conditions of female reproductive anatomy like PCOS, S1P signaling in pregnant WWH and their HIV-negative counterparts with or without sPTB had not previously been investigated. We found that multiple components of S1P signaling, including *SPHK1/2* and *S1PR1/3*, are expressed in the FRT and vary with HIV status and birth outcome. Our results suggest that perturbations in *S1PR1* and *S1PR3* expression may be associated with inflammation related to HIV infection and spontaneous preterm birth and call for further studies of S1P signaling in pregnancy, especially among women living with HIV.

Our studies may have implications for the evolving understanding of the underlying mechanisms of preterm birth and could open avenues to explore novel diagnostics or early indicators of potential preterm birth. By understanding the possible role of S1P signaling in this pregnancy outcome (and in the future, correlating changes in S1P levels between the vagina and placenta during pregnancy), we may be able to apply this knowledge to identify early biomarkers of sPTB. Vaginal swab collection is well-tolerated by women in our study population and easily integrated into routine clinical visits, and a single swab yields sufficient RNA to assess multiple S1P biomarkers in a cost-effective and efficient manner (PCR) within 1–2 days. This suggests that, in theory, screening for S1P-related biomarkers, or other markers of increased risk of preterm birth, via vaginal swab may be a potentially useful tool in resource-limited settings. Moreover, future studies will allow the evaluation of an outcome of long-term ART in WWH—chronic S1P signaling changes in the vagina and placenta and implications for possible adverse birth outcomes.

Defining the landscape of S1P signaling in the female reproductive tract and its association with HIV status and preterm birth is important as we seek to fully understand and prevent adverse birth outcomes and promote long-term well-being and reproductive health for women.

## Figures and Tables

**Table 1:** Participant Data.

	HIV+		HIV−	
	sPTB	Term	sPTB	Term
Participants, n	42	41	42	42
Maternal age	29.0 (25.0–34.0)	30.0 (25.0–32.0)	28.0 (24.0–32.0)	27.0 (24.0–29.0)
Parous	33 (78.6%)	32 (78.0%)	30 (71.4%)	32 (76.2%)
Previous births	2 (1–3)	1 (1–3)	1 (0–3)	1 (1–2)
Prior preterm birth	6 (18.2%)	3 (9.4%)	15 (50.0%)	5 (15.6%)
Prior miscarriage	5 (11.9%)	6 (14.6%)	7 (16.7%)	6 (14.3%)
Prior stillbirth	4 (12.1%)	4 (12.5%)	6 (20.0%)	2 (6.2%)
GA at specimen collection, wks	23.6 (19.0–24.1)	24.1 (22.9–24.6)	24.1 (23.7–24.4)	24.0 (17.9–24.6)
Viral load undetectable ^a^	24 (57.1%)	19 (47.5%)		
Viral load, copies/mL ^a^	39 (0–216)	149 (39–3350)		
CD4 count, cells/µL ^b^	576 (354–789)	533 (321–696)		
ART at baseline (of HIV+)	35 (83.3%)	33 (80.5%)		
Preconceptional ART (of HIV+)	30 (71.4%)	29 (70.7%)		
Hemoglobin < 11g/dL ^c^	6 (18.8%)	13 (34.2%)	3 (11.5%)	3 (11.5%)
Syphilis seropositive	5 (11.9%)	5 (12.2%)	3 (7.1%)	1 (2.4%)
Active tuberculosis	4 (9.5%)	1 (2.4%)	0 (0.0%)	1 (2.4%)
Chronic hypertension	2 (4.8%)	3 (7.3%)	1 (2.4%)	2 (4.8%)
Diabetes outside of pregnancy	0 (0.0%)	0 (0.0%)	0 (0.0%)	0 (0.0%)
Thyroid condition	0 (0.0%)	0 (0.0%)	0 (0.0%)	0 (0.0%)
Education, yrs completed	9.0 (7.0–12.0)	9.0 (7.0–12.0)	11.0 (9.0–12.0)	12.0 (9.0–12.0)
Electricity in household	38 (90.5%)	35 (85.4%)	40 (95.2%)	38 (90.5%)
Flush/pour toilet in household	22 (52.4%)	20 (48.8%)	23 (54.8%)	23 (54.8%)
Neither married nor cohabiting	9 (21.4%)	5 (12.2%)	6 (14.3%)	9 (21.4%)
Alcohol use during pregnancy	4 (9.5%)	4 (9.8%)	5 (11.9%)	2 (4.8%)

^a^ N = 82 (42 HIV+/sPTB, 40 HIV+/Term); ^b^ N = 80 (42 HIV+/sPTB, 38 HIV+/Term); ^c^ N = 122 (32 HIV+/sPTB+, 38 HIV+/Term, 26 HIV−/sPTB, 26 HIV−/Term).

## Data Availability

The raw data supporting conclusions presented in this study are available on request from the corresponding authors due to privacy and ethical reasons.

## Supplementary Materials

The following supporting information can be downloaded at: https://www.mdpi.com/article/10.3390/v18050559/s1, Figure S1: Gestational age at sample collection vs S1P biomarkers.; Figure S2: Correlation of *SPHK1/2* and *S1PR1/3* with viral load.; Figure S3: Correlation of *SPHK1/2* and *S1PR1/3* with CD4 count.
