# Supplementary material for: Sphingosine-1-Phosphate Receptor and Kinase Expression in the Reproductive Tract Is Associated with HIV Infection and Preterm Birth in a Cohort of Pregnant Women in Zambia
Source: Viruses. 2026 May 14;18(5):559. doi: 10.3390/v18050559 (PMC13211646; doi:10.3390/v18050559)
Supplement: Supplementary file 1 [file viruses-18-00559-s001.zip › viruses-4204616-supplementary.pdf]

Supplemental Figure 1

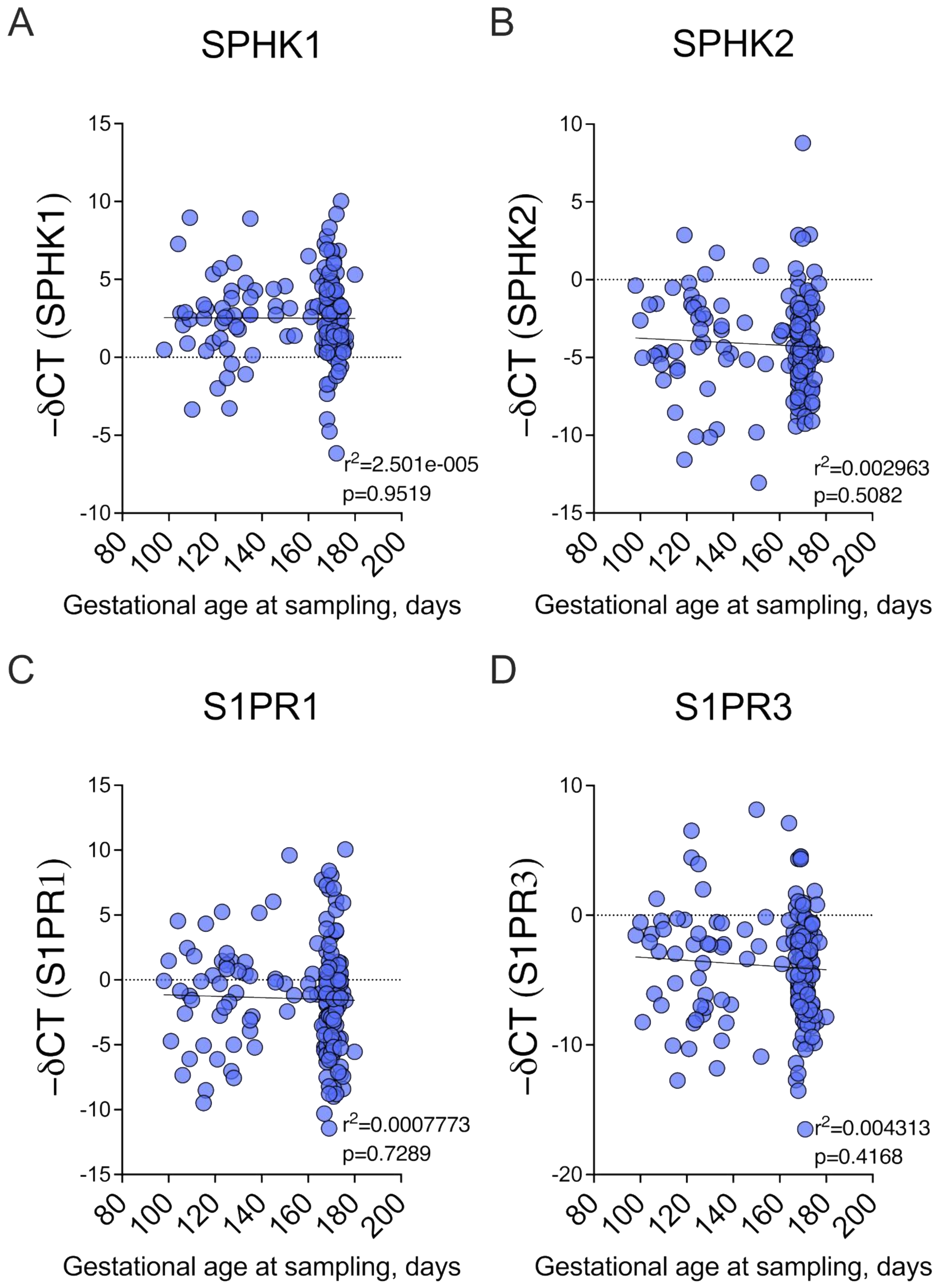

Supplemental Figure 1: Gestational age at sample collection vs S1P biomarkers. Days of gestation at sample (vaginal swab) collection vs. A. −ΔCT *SPHK1*, B. −ΔCT *SPHK2*, C. −ΔCT *S1PR1*, and D. −ΔCT *S1PR3*. Simple linear regression resulted in slopes for gestational age at collection vs −ΔCT *SPHK1*/*2* and *S1PR1*/*3* that were not statistically significantly different than zero.

Supplemental Figure 2

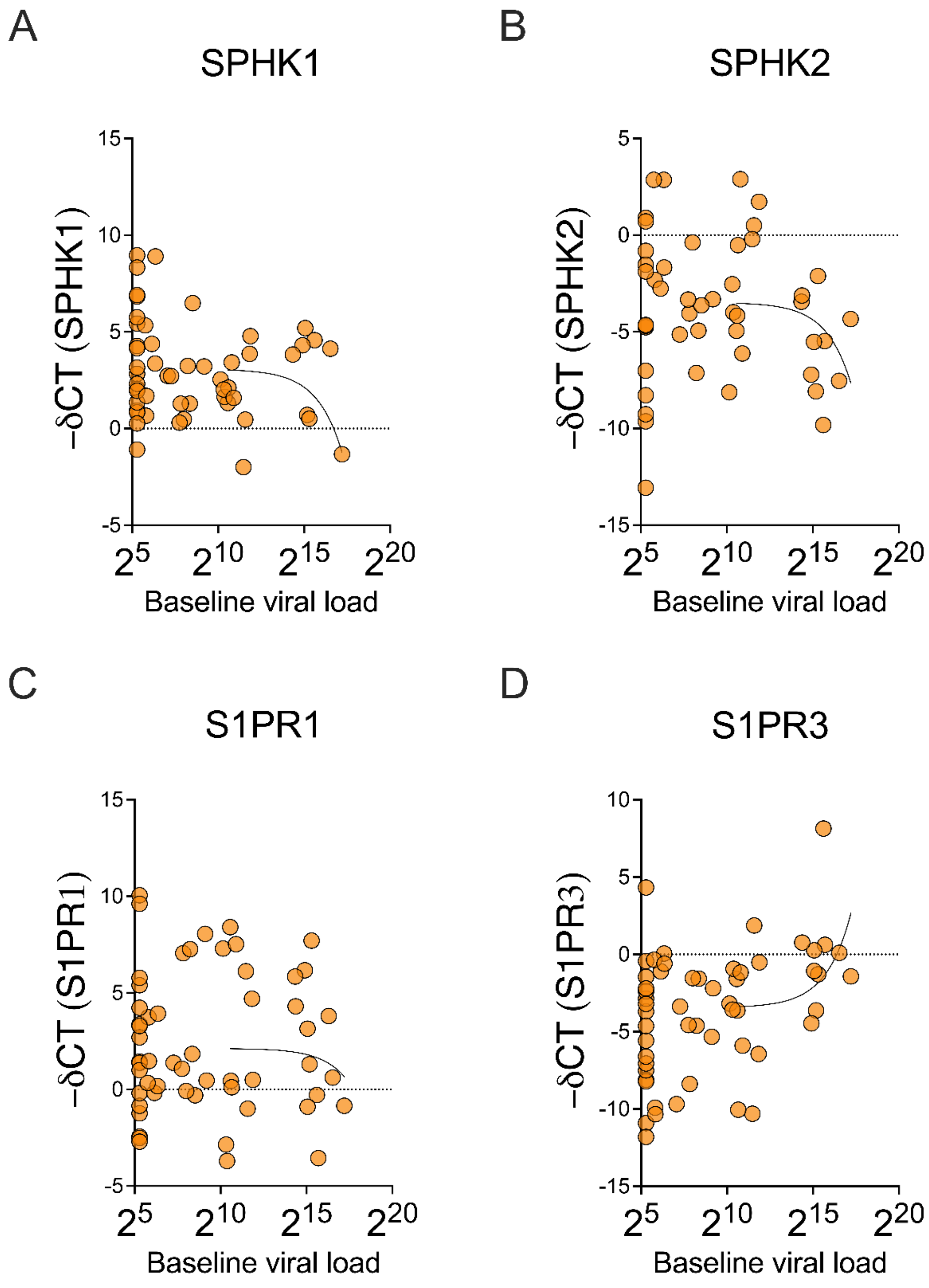

Supplemental Figure 2. Correlation of *SPHK1/2* and *S1PR1/3* with viral load. A. *SPHK1*, B. *SPHK2*, C. *S1PR1* and D. *S1PR3* were examined for a potential correlation with viral load at baseline. Simple linear regression resulted in slopes for viral load vs −ΔCT *SPHK1/2* and *S1PR1/3* that were not statistically significantly different than zero.

Supplemental Figure 3

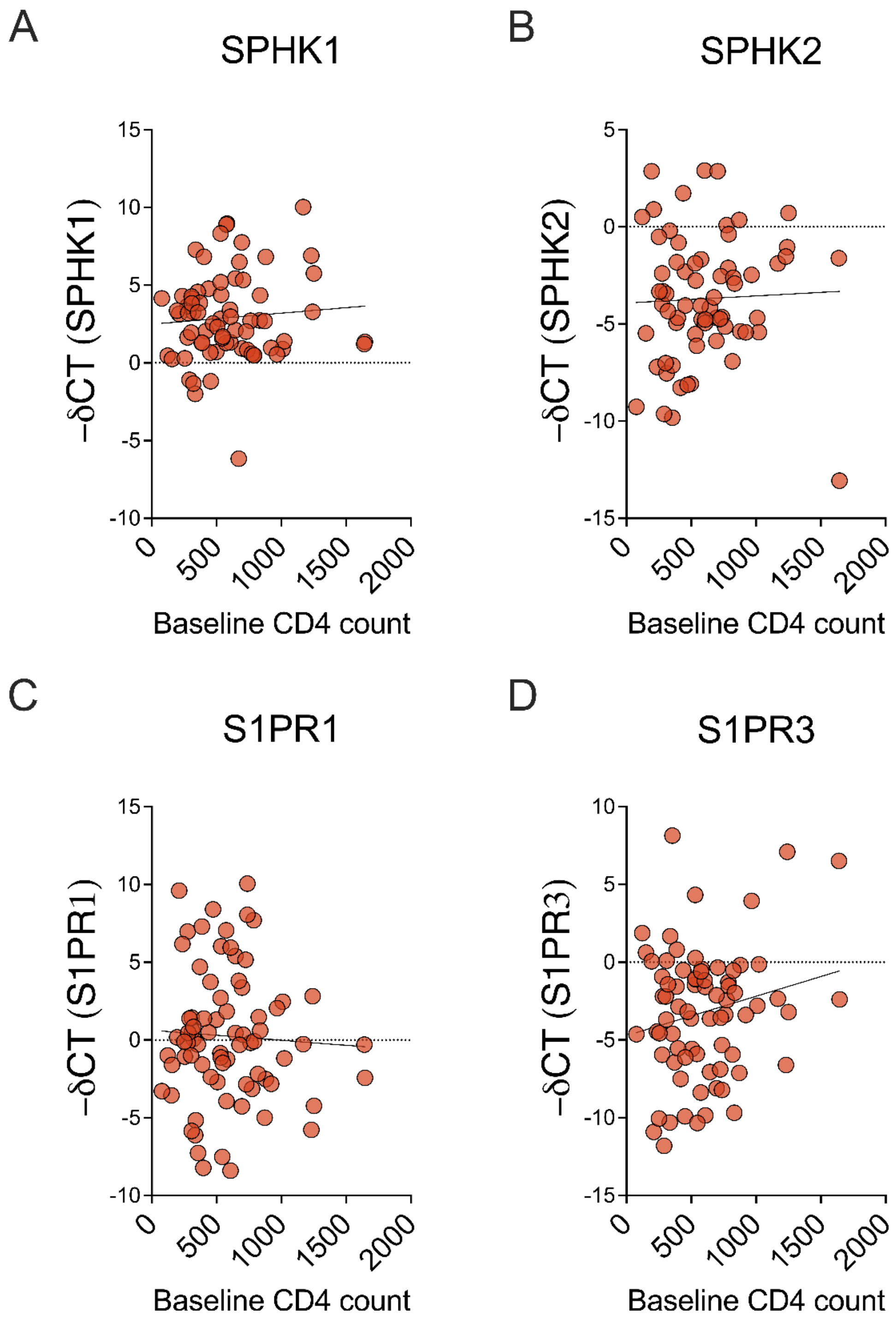

Supplemental Figure 3. Correlation of *SPHK1*/*2* and *S1PR1*/*3* with CD4 count. A. *SPHK1*, B. *SPHK2*, C. *S1PR1* and D. *S1PR3* were examined for a potential correlation with CD4 count at baseline. Simple linear regression resulted in slopes for viral load vs −ΔCT *SPHK1*/*2* and *S1PR1*/*3* that were not statistically significantly different than zero.
